# Real-life analysis of neoadjuvant-therapy-associated benefits for pathological complete response and survival in early breast cancer patients - role of trastuzumab in HER2+ BC and platinum in TNBC

**DOI:** 10.3389/fonc.2022.1022994

**Published:** 2023-01-24

**Authors:** Wei-Pang Chung, Chun-Ting Yang, Shuen-Ru Yang, Ching-Yen Su, Hsin-Wei Su, Shang-Yun Liu, Huang-Tz Ou

**Affiliations:** ^1^ Department of Oncology, National Cheng Kung University Hospital, College of Medicine, National Cheng Kung University, Tainan, Taiwan; ^2^ Center of Applied Nanomedicine, National Cheng Kung University, Tainan, Taiwan; ^3^ Institute of Clinical Pharmacy and Pharmaceutical Sciences, College of Medicine, National Cheng Kung University, Tainan, Taiwan; ^4^ Medical Division, Roche Products Ltd., Taipei, Taiwan; ^5^ Department of Pharmacy, College of Medicine, National Cheng Kung University, Tainan, Taiwan

**Keywords:** early breast cancer, HER2-positive breast cancer, triple-negative breast cancer, trastuzumab, platinum, neoadjuvant therapy

## Abstract

**Background:**

Neoadjuvant therapy, which aims to achieve a pathological complete response (pCR) for better overall survival (OS) has several advantages for patients with early breast cancer (eBC) and subtypes of HER2-positive (HER2+) and triple-negative breast cancer (TNBC). However, there has been no large-scale real-world investigation on the clinical outcomes associated with trastuzumab-based and platinum-based neoadjuvant treatments for patients with HER2+ and TNBC, respectively.

**Material and methods:**

Taiwan Cancer Registry and National Health Insurance Research Database were utilized in this study. Patients diagnosed with clinically lymph-node-positive (LN+) HER2+ or TNBC were identified for analysis. Logistic regression and Cox proportional hazard models were employed to estimate the adjusted odds ratios (aOR) of achieving pCR and adjusted hazard ratios (aHR) of overall survival associated with treatment agents, respectively.

**Results:**

A total of 1,178 HER2+ eBC and 354 early TNBC patients were identified, respectively. Neoadjuvant trastuzumab significantly increased the pCR rates by 3.87-fold among HER2+ patients. Trastuzumab-associated survival benefit was found in HER2+ patients who achieved pCR (aHR [95% CI]: 0.30 [0.11-0.84]) but not in those without pCR (1.13 [0.77-1.67]). Among the TNBC patients, platinum was associated with a 1.6-fold increased pCR rate; however, it did not improve OS regardless of pCR status.

**Conclusions:**

Trastuzumab improved pCR and OS for patients with HER2+ subtype. Using platinum agents for TNBC patients increased pCR rates but was not linked to better survival. Optimal neoadjuvant anti-HER2 therapy for patients with HER2+ eBC and the introduction of novel therapy for patients with TNBC should be considered.

## Introduction

Neoadjuvant therapy offers some advantages for patients with early breast cancer (eBC), including down-sizing of tumors, an opportunity for less extensive surgical resection, and outcome assessment and risk stratification based on results of pathological status (1). Attaining a pathological complete response (pCR), which refers to the absence of invasive cancer in the breast and axillary lymph nodes, has been widely discussed for neoadjuvant therapy. With adequate treatment, pCR rates are higher for human epidermal growth factor receptor 2-positive (HER2+) and triple-negative breast cancer (TNBC) subtypes compared to the quite low rates for the hormone receptor (HR)-positive/HER2-negative subtype (2). The achievement of pCR in HER2+ and TNBC patients has been associated with better outcomes, including event-free survival and overall survival (OS) (2–4).

For patients with HER2+ eBC, anti-HER2 monoclonal antibodies with chemotherapy are the standard of care in neoadjuvant settings. Trastuzumab, the first anti-HER2 monoclonal antibody, significantly enhances the pCR rates for the HER2+ population (5–7) and is widely used in current practice. Although adding pertuzumab to a trastuzumab-based regimen (referred to as dual HER2 blockade) has led to higher pCR rates (8–10), pertuzumab is not commonly used in several countries due to its high drug acquisition cost. Therefore, trastuzumab remains the primary treatment strategy for HER2+ patients in these countries. However, a limited number of studies in real-world settings have examined whether the trastuzumab-associated benefits for pCR translate to lifelong survival outcomes (i.e., OS).

For TNBC, the benefit of adding platinum agents, which have cytotoxic DNA-damaging effects, to neoadjuvant therapy to enhance pCR rates has been observed in trial (11–13) and real-world populations (14, 15), while an insignificant difference in OS associated with the use of platinum-based therapy has been reported by retrospective studies (15–17). However, the interpretation of previous findings should be done with caution because 1) a single-center design with a small number of platinum users may attenuate statistical power and limit the generalizability of study findings (14, 17), 2) the lack of adjustment for potential confounding effects attributable to patient baseline and comorbid conditions may have biased the study estimates (15, 17), and 3) analyses conducted without considering patient pCR status may lead to mixed estimates that cannot be differentiated for pCR and non-pCR populations (15–17). The use of platinum-based neoadjuvant regimens in TNBC remains inconclusive in the scientific community, as evident in international clinical practice guidelines (18).

Against this background, the present study assessed the real-world survival outcomes associated with specific neoadjuvant treatments among high-risk eBC patient populations (trastuzumab and platinum agents for HER2+ and TNBC populations, respectively) using large-scale real-world databases to allow long-term observation of survival outcomes as well as rigorous analytic strategies to ensure the validity and practicability of study findings for daily treatment and health policy decision-making.

## Material and methods

### Data source

The Taiwan Cancer Registry (TCR) and the National Health Insurance Research Database (NHIRD), two nationally representative data sources in Taiwan, were utilized in this study. The TCR is a nationwide cancer registry database that covers 97% of Taiwanese patients with confirmed cancers and provides essential information for newly diagnosed cancer cases such as the clinical and pathological tumor, node, and metastasis staging at cancer diagnosis (19). The NHIRD comprises the longitudinal medical records of enrollees in Taiwan’s National Health Insurance (NHI) program, which covers around 99% of residents in Taiwan, including the disease diagnoses and treatment procedures in ambulatory, inpatient, and emergency departments of medical institutions, and prescription drugs in pharmacy claims data (20). The patient-level data from the TCR and the NHIRD were linked by individual, encrypted, and de-identified numbers from the Health and Welfare Data Science Center in Taiwan. This study was approved by the Institutional Review Board of National Cheng Kung University (A-EX-106-029).

### Cohort identification

First, we included patients aged ≥ 18 years with *de novo* eBC during 2011-2016 and without any malignancy history before BC diagnosis to avoid potential confounding from prior cancers. Patients without available information for measuring the date of BC diagnosis and its subtypes (i.e., HR+ & HER2-, HER2+, and TNBC) and determining the eligibility of receiving neoadjuvant therapy (i.e., clinical staging of N0 and T0/T1) were excluded to minimize potential misclassification bias. Second, among patients who were eligible for neoadjuvant therapy, those having the clinical staging of lymph-node-positive (LN+) were further identified under consideration of available neoadjuvant drugs. To reduce the heterogeneity of the study subjects, patients who had not received BC surgery within 1 year of BC diagnosis were excluded. Third, patients who received neoadjuvant therapy and whose pCR status was available were identified. The initiation date of neoadjuvant therapy was defined as the index date. Lastly, we only included patients with the HER2+ or TNBC subtype in the study cohort for analyzing treatment-associated outcomes (i.e., trastuzumab and platinum exposure during the period of receiving neoadjuvant therapy for HER2+ and TNBC patients, respectively).

**Figure 1:**
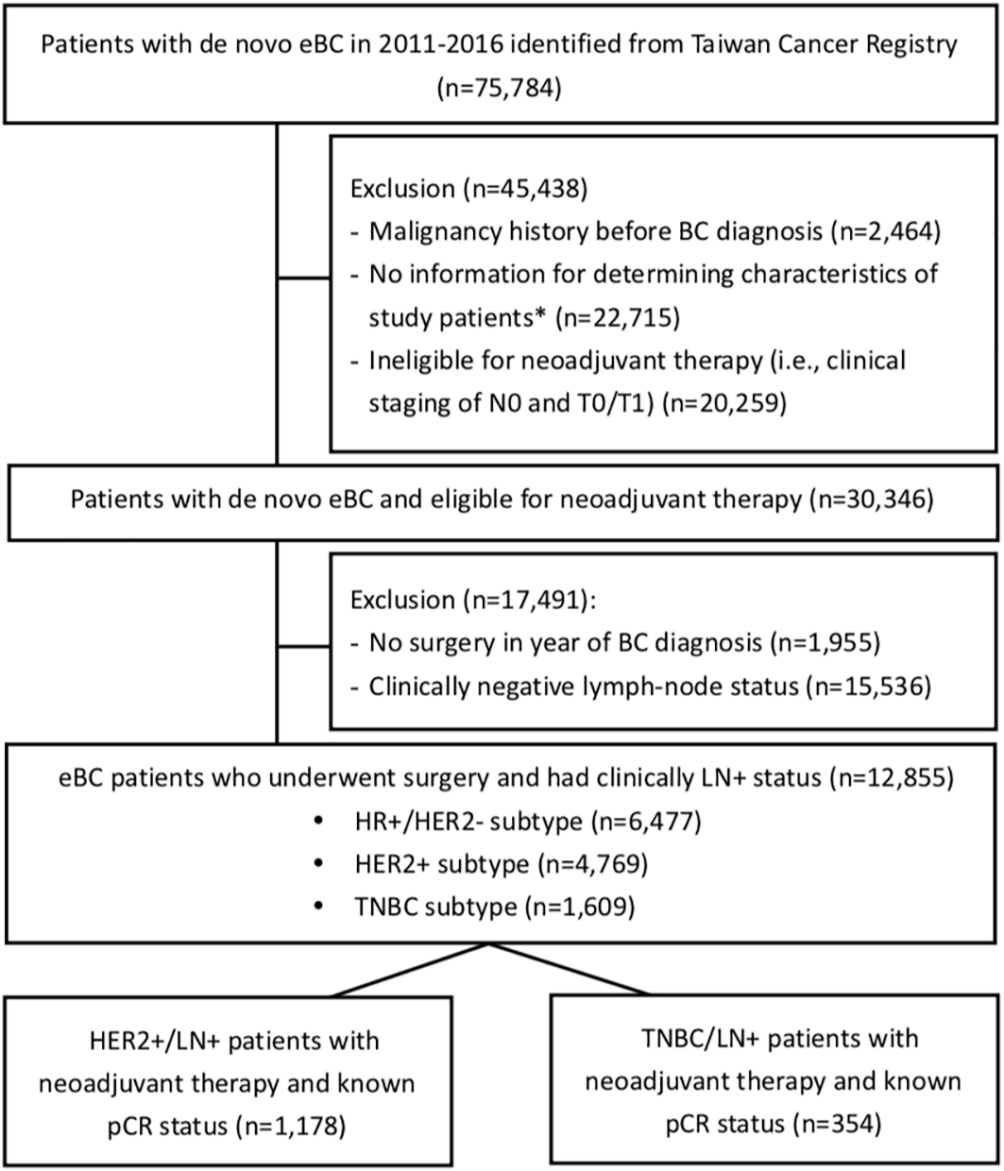
Flowchart of cohort selection. eBC, early breast cancer; BC, breast cancer; pCR, pathological complete response; HR, hormone receptor; HER2, human epidermal growth factor receptor-2; TNBC, triple-negative breast cancer; LN+, lymph-node positive. *The characteristics included the clinical staging of BC, subtype of BC (i.e., HR+ & HER2-, HER2+, and TNBC), diagnosis date of BC, and the eligibility of receiving neoadjuvant therapy (i.e., clinical N or T staging as the eligibility of receiving neoadjuvant therapy).

The study subjects were followed from the first date of neoadjuvant therapy (i.e., index date) until death, loss of follow-up, or the end of data availability of the NHIRD (i.e., December 31, 2018), whichever came first. The cohort selection is detailed in **Figure 1**.

### Study variables

Information on neoadjuvant therapy and exposure to the given treatment agents were obtained from the NHIRD and defined using the Anatomical Therapeutic Chemical Classification System (e.g., L01XC03 for trastuzumab and L01XA01/L01XA02 for platinum agents). The treatment-associated outcomes, pCR (defined as ypT0/is ypN0) and survival status of study subjects, were confirmed by the TCR and the Cause of Death File in the NHIRD, respectively. Patients’ baseline comorbidities in the year prior to or at the index date were measured based on the disease diagnosis records in the NHIRD using the International Classification of Diseases, Ninth Revision, Clinical Modification and the International Classification of Diseases, Tenth Revision, Clinical Modification, and are presented according to the Charlson Comorbidity Index (CCI) (21).

### Statistical analysis

The adoption rate of neoadjuvant therapy was calculated as the number of patients who had undergone neoadjuvant therapy divided by the number of patients who were eligible for neoadjuvant therapy. The Cochran-Armitage test was used to evaluate the time trend of adoption rates in the period 2011-2016. The descriptive statistics of patients’ baseline characteristics were measured and are presented as mean values with standard deviations for continuous variables and frequencies with proportions for categorical variables. In addition, the differences in patients’ baseline characteristics between study comparison groups (i.e., pCR versus no pCR, trastuzumab versus no trastuzumab, platinum versus no platinum) were evaluated using Student’s *t*-test and the chi-squared test for continuous and categorical variables, respectively. The pCR rate was calculated as the number of patients with pCR divided by the number of total study subjects. The OS values of patients with and without pCR are plotted separately using Kaplan-Meier (KM) curves. The association between pCR achievement and patients’ OS was analyzed using Cox proportional hazard models, which modeled the time-to-death of individual subjects as a function of treatment exposure (i.e., pCR versus no pCR) with adjustment for patients’ baseline characteristics. The relative hazards of the all-cause death of patients with pCR versus those of patients without pCR are presented as adjusted hazard ratios (aHRs) with the associated 95% confidence intervals (CIs).

Further, the clinical effectiveness of achieving pCR associated with individual treatment agents, namely trastuzumab for HER2+ patients and platinum agents for TNBC patients, was assessed using logistic regression models and is presented as adjusted odds ratios (aORs) with the associated 95% CIs. Survival analyses (i.e., Kaplan-Meier curves and Cox models) were also applied to evaluate the trastuzumab- and platinum-associated survival outcomes for HER2+ and TNBC patients, respectively. All *p*-values were two-tailed; values of less than 0.05 were considered statistically significant. The above statistical analyses were conducted using SAS 9.4 software (SAS Institute, Inc., Cary, NC, USA).

## Results

**Figure 2:**
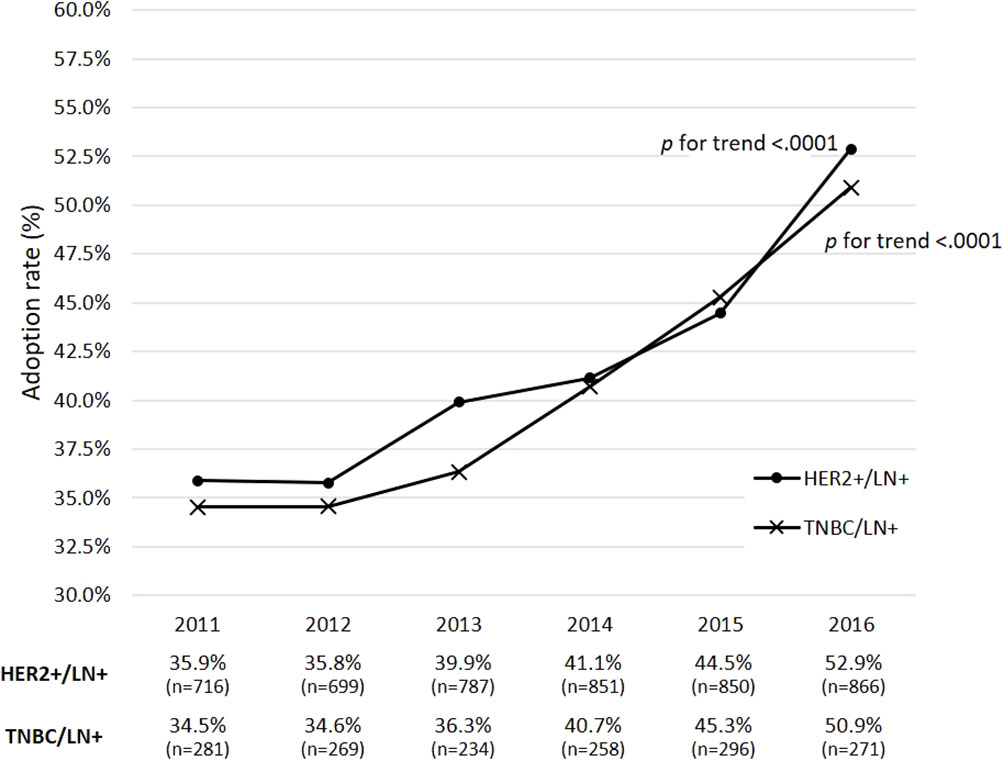
Adoption rates of receiving neoadjuvant therapy during 2011-2016 among eBC patients with clinically positive lymph-node status. *p* refers to the *p*-value for a time trend; a *p*-value of less than 0.05 indicates a significant change of adoption rates of neoadjuvant therapy over time. eBC, early breast cancer; BC, breast cancer; pCR, pathological complete response; HR, hormone receptor; HER2, human epidermal growth factor receptor-2; TNBC, triple-negative breast cancer; LN+, lymph-node positive.

Among the 30,346 eBC patients who were eligible for neoadjuvant therapy during 2011-2016, 12,855 were LN+ and received breast-cancer-related surgeries within 1 year of BC diagnosis (**Figure 1**). Those with HER2+ and TNBC subtypes accounted for 37.1% (n=4,769) and 12.5% (n=1,609) of the 12,855 subjects, respectively. **Figure 2** shows that the rates of receiving neoadjuvant treatment among HER2+ and TNBC patients both significantly increased over time (*p*-values for time trend <0.0001). Overall, for the period 2011-2016, a total of 1,178 HER2+ and 354 TNBC patients who received neoadjuvant therapy and had a known pCR status were identified as study patients for the analysis of treatment-associated outcomes. Among selected study subjects, docetaxel, cyclophosphamide and epirubicin were the most commonly prescribed chemotherapy agents for both HER2+ and TNBC patients (**Supplementary Figure 1**).

**Table 1** provides the baseline characteristics of study patients stratified by pCR achievement and eBC subtype. Generally, the cohort was mainly composed of patients with clinical stage 2B and 3A (i.e., around 70%). The demographic characteristics (i.e., age, gender) and baseline comorbidities (i.e., CCI scores) were similar between patients with and without pCR, except for a younger age for patients with pCR versus those without pCR among TNBC subjects. However, the proportions of patients stratified by the clinical stage of BC were significantly different between patients with and without pCR in both HER2+ and TNBC subjects.

**Table 1:** Baseline patient characteristics stratified by the subtype of BC and status of pCR.

Subtypes	HER2+ with positive nodal status		TNBC with positive nodal status	
	With pCR (n=460)	Without pCR (n=718)	With pCR (n=128)	Without pCR (n=226)
Age, years (mean ± SD)*	52.16 ± 10.26	51.71 ± 10.42	8.38 ± 10.29	51.77 ± 11.18
**Female (%)**	460 (100%)	717 (99.86%)	128 (100%)	226 (100%)
Year of BC diagnosis (%)*				
**2011**	20 (4.35%)	85 (11.84%)	7 (5.47%)	27 (11.95%)
**2012**	39 (8.48%)	91 (12.67%)	18 (14.06%)	31 (13.72%)
**2013**	75 (16.30%)	87 (12.12%)	15 (11.72%)	21 (9.29%)
**2014**	87 (18.91%)	149 (20.75%)	17 (13.28%)	41 (18.14%)
**2015**	97 (21.09%)	144 (20.06%)	27 (21.09%)	51 (22.57%)
**2016**	142 (30.87%)	162 (22.56%)	44 (34.38%)	55 (24.34%)
BR grade (%)				
**High**	165 (35.87%)	282 (39.28%)	19 (14.84%)	51 (22.57%)
**Low**	89 (19.35%)	147 (20.47%)	52 (40.63%)	98 (43.36%)
**Others**	206 (44.78%)	289 (40.25%)	57 (44.53%)	77 (34.07%)
Clinical stage (%)*				
**Stage 2A**	29 (6.30%)	20 (2.79%)	4 (3.13%)	8 (3.54%)
**Stage 2B**	172 (37.39%)	259 (36.07%)	67 (52.34%)	77 (34.07%)
**Stage 3A**	151 (32.83%)	223 (31.06%)	37 (28.91%)	76 (33.63%)
**Stage 3B**	49 (10.65%)	117 (16.30%)	6 (4.69%)	33 (14.60%)
**Stage 3C**	59 (12.83%)	99 (13.79%)	14 (10.94%)	32 (14.16%)
Medical history				
**CCI = 0 (%)**	325 (70.65%)	538 (74.93%)	101 (78.91%)	167 (73.89%)
**CCI = 1 (%)**	84 (18.26%)	125 (17.41%)	23 (17.97%)	39 (17.26%)
**CCI ≥ 2 (%)**	51 (11.09%)	55 (7.66%)	4 (3.13%)	20 (8.85%)

BC, breast cancer; pCR, pathological complete response; HER2, human epidermal growth factor receptor-2; TNBC, triple-negative breast cancer; BR grade, Nottingham modification of the Bloom-Richardson grading system; CCI, Charlson Comorbidity Index.

*There were significant differences (*p*<0.05) in the year of BC diagnosis and clinical staging between HER2+ patients with pCR and those without pCR. For patients with TNBC, the age at BC diagnosis and clinical staging of those with pCR was statistically different compared to those without pCR.

**Figure 3:**
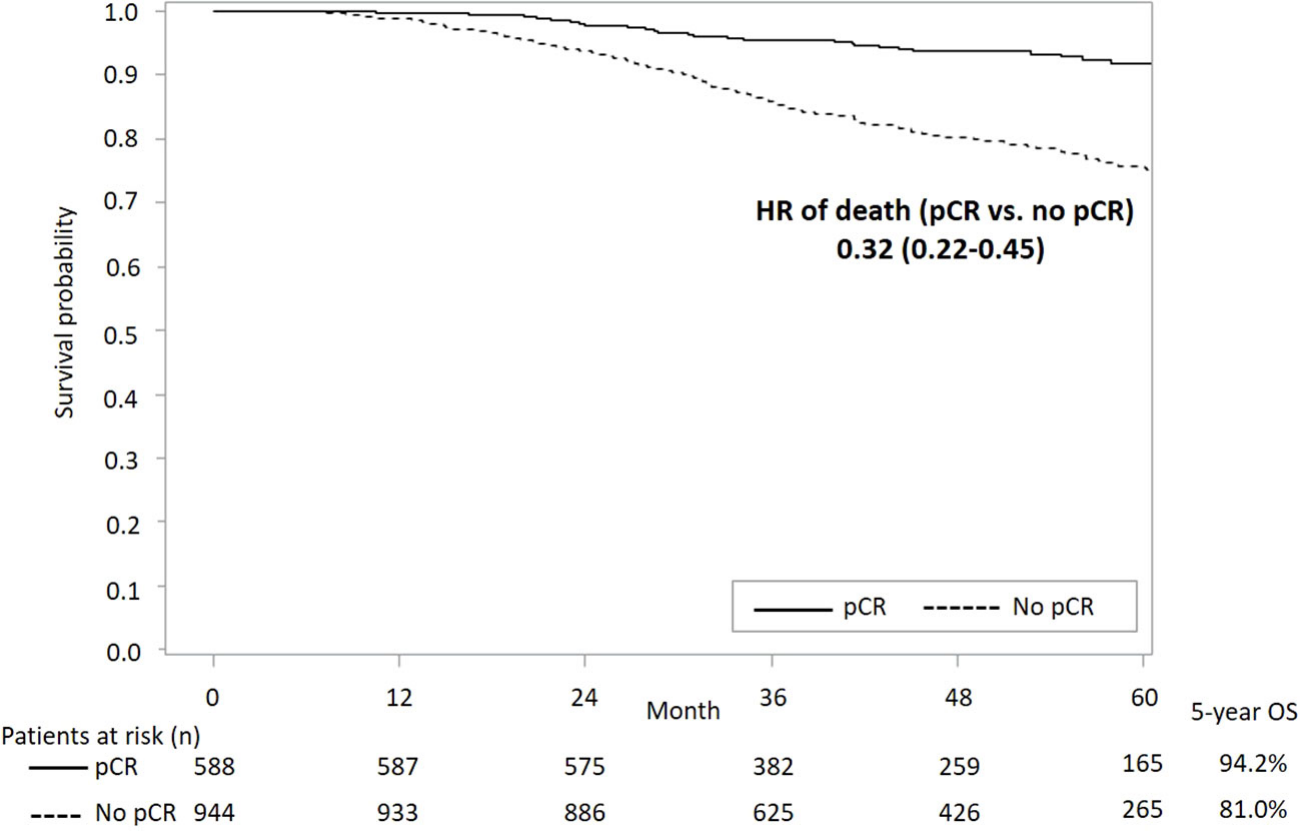
KM curves of OS stratified by status of pCR in overall study population. KM, Kaplan-Meier; OS, overall survival; pCR, pathological complete response; HR, hazard ratio.

**Figure 4:**
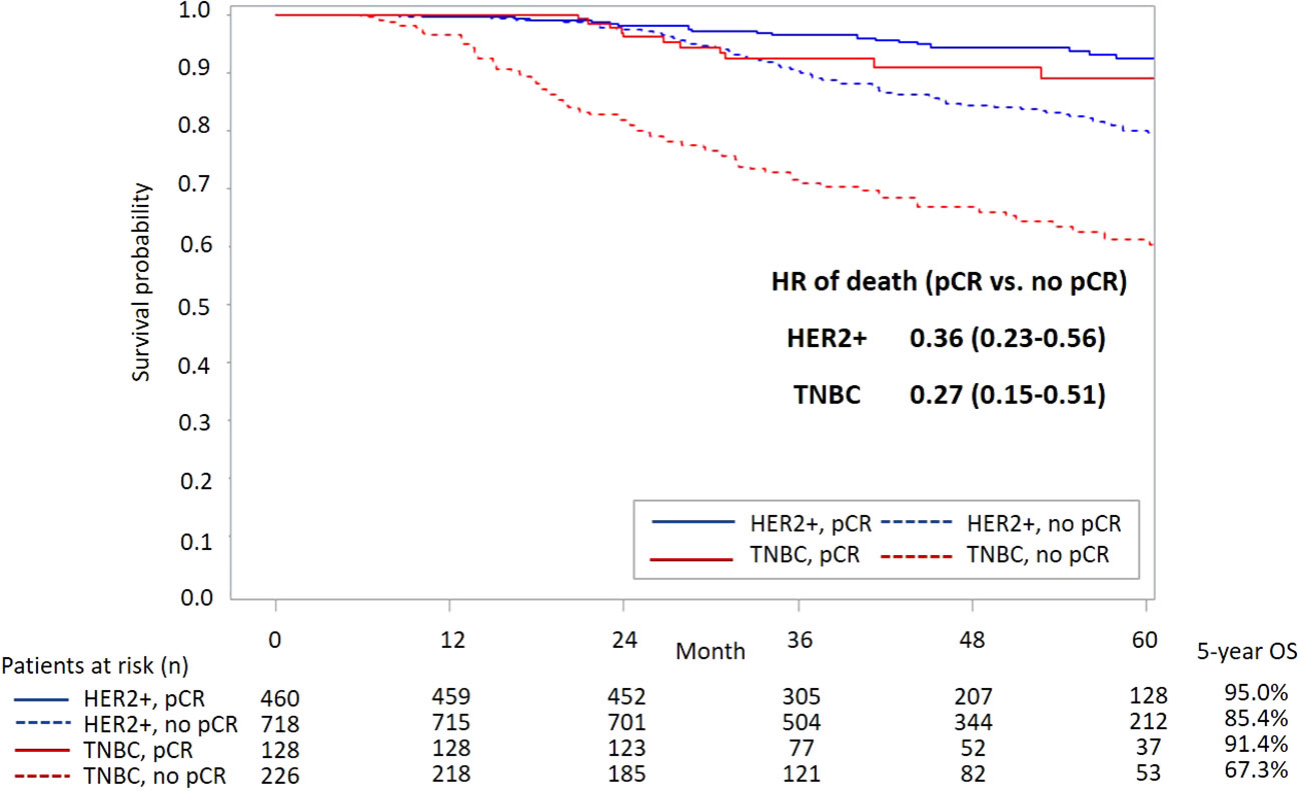
KM curves of OS stratified by status of pCR and subtype. KM, Kaplan-Meier; OS, overall survival; pCR, pathological complete response; HR, hazard ratio; HER2, human epidermal growth factor receptor-2; TNBC, triple-negative breast cancer.

**Figure 3** presents survival curves stratified by the pCR status in the overall study population. It shows a significantly lower all-cause mortality among patients with pCR versus those without pCR (aHR [95% CI]: 0.32 [0.22-0.45]). A better survival outcome with pCR achievement was also observed in patients with either subtype HER2+ or TNBC (**Figure 4**).

During 2011-2016, the rates of patients with exposure to trastuzumab and platinum among HER2+ and TNBC patients, respectively, both increased with time (**Supplementary Figures 2**, **3**). The baseline characteristics of HER2+ patients with/without exposure to trastuzumab and TNBC patients with/without exposure to platinum are provided in **Supplementary Tables 1**, **2**, respectively. **Table 2** shows the pCR rates and treatment-associated ORs for patients with pCR. Among HER2+ patients, the pCR rates were 46.98% and 14.08% for patients with and without trastuzumab treatment, respectively. Patients who were exposed to trastuzumab had a 3.87-fold increase in the odds of achieving pCR compared with those without trastuzumab exposure (aOR [95% CI]: 4.87 [3.34-7.10]). For TNBC patients, the pCR rates were 49.59% and 29.00% for patients with and without platinum treatment, respectively. The exposure to platinum agents was associated with a 1.6-fold increase in the odds of pCR achievement (aOR [95% CI]: 2.60 [1.59-4.26]).

**Table 2:** Treatment-associated odds ratios of achieving pCR and hazard ratios of all-cause death (reference case: no treatment)*.

Subtypes of BC	HER2+ with positive nodal status		TNBC with positive nodal status	
	Trastuzumab (n=894)	No trastuzumab (n=284)	Platinum (n=123)	No platinum (n=231)
**pCR rate**	46.98%	14.08%	49.59%	29.00%
**Odds ratios (95% CI) of achieving pCR^†^**	4.87 (3.34, 7.10)		2.60 (1.59, 4.26)	
Hazard ratios (95% CI) of all-cause death^†^				
**Patients with pCR**	0.30 (0.11-0.84)		0.49 (0.12-2.10)	
**Patients without pCR**	1.13 (0.77-1.67)		0.74 (0.43-1.27)	

pCR, pathological complete response; BC, breast cancer; HER2, human epidermal growth factor receptor-2; TNBC, triple-negative breast cancer; CI, confidence interval.

*The reference cases for odds ratios and hazard ratios for patients with HER2+ and TNBC were those without trastuzumab exposure and those without platinum exposure during the period of receiving neoadjuvant therapy, respectively.

^†^The odds ratios and hazard ratios were adjusted for the imbalance of patients’ baseline characteristics between those with and without exposure to trastuzumab for HER2+ patients (**Supplementary Table 1**) and between those with and without exposure to platinum for TNBC patients (**Supplementary Table 2**).

**Table 2** and the **Supplementary Figures 4**-**7** show that among HER2+ patients with pCR achievement, patients who received trastuzumab had a better survival outcome compared with those without trastuzumab exposure (aHR [95% CI]: 0.30 [0.11-0.84]; **Supplementary Figure 4**); however, no survival benefit with trastuzumab treatment was observed among HER2+ patients without pCR (aHR [95% CI]: 1.13 [0.77-1.67]; **Supplementary Figure 5**). For patients with TNBC, exposure to platinum agents was associated with an insignificantly lower all-cause mortality regardless of patients’ pCR status (**Supplementary Figures 6**, **7**).

## Discussion

This real-world analysis of a large and nationwide eBC patient population with a long-term follow-up supports that the survival benefit of achieving pCR following neoadjuvant therapy among eBC patients, which has been mainly reported for trial settings (2), can be extended to real-world practice, especially for HER2+ and TNBC patients with clinically positive lymph node status, who represent high risk cases of tumor recurrence with unmet need for novel treatments. This cohort study also demonstrated that adoption rates of neoadjuvant therapy among patients with HER2+ and TNBC increased with time, which was in line with the global trend. We further showed that the combination of trastuzumab with neoadjuvant chemotherapy as standard care under current national reimbursement scheme among HER2+ patients was significantly associated with better pCR rates and subsequently enhanced survival outcomes of patients. This confirms the important role of targeted agents and suggests that tailored anti-HER2 target therapy should be encouraged to optimize patients’ survival. Moreover, given the controversy regarding the survival outcomes obtained by adding platinum agent to standard neoadjuvant chemotherapy for TNBC patients, our real-life analyses showed a significantly enhanced pCR achievement of platinum-based neoadjuvant chemotherapy; however, this short-term benefit for pCR was not further linked to the overall survival benefit for patients. Novel treatments in neoadjuvant settings for the TNBC population thus require future research.

### Association between pCR and OS in HER2+ and TNBC patients

In this real-world cohort, pCR achievement was associated with 64% and 73% reductions in all-cause mortality among BC patients with HER2+ and TNBC subtypes, respectively. These reductions are slightly lower than previously reported meta-analysis findings, especially for the TNBC patients (2, 4). For example, the large-scale CTNeoBC pooled analysis of trial populations revealed 66% and 84% reductions in mortality associated with attaining pCR in HER2+ and TNBC populations, respectively (2). Huang et al. reported an 81% reduction in death risk among TNBC patients who achieved pCR versus those who did not (4).

Note that direct comparisons between the present study results and previous findings should be conducted with caution due to the following study differences. First, unlike the trial populations included in previous meta-analyses, our study focused on real-world eBC populations who had more diverse patient profiles in terms of more complicated clinical characteristics and more varied treatment histories, resulting in potentially increased risks of clinical events (e.g., death). Second, the present study was conducted in a clinical setting with treatments mainly covered by the NHI program. Third, only patients with clinically LN+ status were included in this study, which led to a higher risk of mortality compared with that in previous studies. We only collected data from LN+ patients because trastuzumab is not a reimbursed neoadjuvant item for patients with negative-lymph-node status in Taiwan and taxanes could not be used for the TNBC population without lymph node metastasis before September 2012. Although this cohort enrolled patients with higher risk of recurrence and no assistance from novel agents, we still demonstrated that achieving pCR by neoadjuvant treatment was associated with better survival outcomes compared to those of patients who did not achieve pCR (**Figures 3**, **4**). Nevertheless, to enhance patients’ pCR rates among HER2+ eBC and TNBC populations, optimizing neoadjuvant therapy using effective treatment agents remains crucial.

### Effect of trastuzumab on pCR and overall survival among patients with HER2+ eBC

In neoadjuvant settings, the benefit of trastuzumab for enhancing pCR rates among HER2+ eBC patients has been reported worldwide (5, 6). However, few real-world studies have focused on the long-term survival outcomes following trastuzumab-related pCR achievement. A retrospective study of stage II and III HER2+ eBC populations found that adding trastuzumab to neoadjuvant therapy improved the OS in HER2+/hormone receptor-positive eBC patients (aHR [95% CI]: 0.36 [0.13-0.97]) but the treatment effects in patients who achieved pCR was not further analyzed due to the very limited number of cases; this may limit the application of study findings in real-world practice because a patient’s response (i.e., pCR) to neoadjuvant therapy is crucial in treatment decision-making (22).

By using a large Asian patient cohort that allowed comprehensive and detailed stratification analyses, we corroborated a significantly higher pCR rate following trastuzumab therapy (aOR [95% CI]: 4.87 [3.34-7.10]) and further showed the apparent survival benefit of trastuzumab treatment among patients with pCR achievement (aHR [95% CI]: 0.30 [0.11-0.84]). Accordingly, we infer that achieving pCR using anti-HER2 target therapy (e.g., trastuzumab) is promising for favorable survival outcomes of HER2+ patients.

Regarding patients who did not achieve pCR following trastuzumab-based neoadjuvant chemotherapy, trastuzumab may offer little survival benefit compared with chemotherapy alone. A possible solution is to incorporate other anti-HER2 targeted agents such as pertuzumab to increase the pCR rate (8–10). Another strategy is to rescue patients with residual invasive disease by applying the antibody-drug conjugate, trastuzumab emtansine (T-DM1), instead of continuing trastuzumab treatment in the adjuvant setting (23). Hence, the strategic utilization of these anti-HER2 agents is important to optimize patients’ survival.

### Effect of platinum on pCR and OS among patients with TNBC

The outcomes of recurrent TNBC patients are poor, with a 12-month OS rate that is as low as 38.1% in an American cohort and 28% in a Peru cohort (24, 25). For years, serving as adjuvant medications, only capecitabine in the non-pCR population and olaparib in patients with germline *BRCA1/2* mutations improved TNBC patients’ prognosis. Therefore, neoadjuvant treatment remains an important strategy for achieving pCR, which is a key treatment goal for these patients (26, 27). An apparent difference in the OS following neoadjuvant treatment between TNBC patients with and without attaining pCR was shown in this study, with an aHR of 0.27 (CI: 0.15-0.51) for favoring the pCR population. Unfortunately, an increased chance of achieving pCR (adjusted OR: 2.60, CI: 1.59-4.26) following platinum-based therapy did not translate to a long-term survival benefit among both the pCR and non-pCR populations, despite great effort being made in this study to address the methodology concerns that have been raised in previous real-world studies (14–17). That is, we adjusted for potential confounding effects attributable to patient baseline conditions using multivariate regression models and stratified the analyses by patients’ pCR status to generate precise study estimates for the pCR and non-pCR populations separately.

Considering that the platinum-associated benefit for pCR did not translate to better survival outcomes in both trial (11, 12, 28) and real-world TNBC populations (15–17), novel agents such as immunotherapy should be considered in the neoadjuvant setting for this population (29, 30). Specifically, the KEYNOTE-522 trial as an example demonstrated that the addition of pembrolizumab, an immune checkpoint inhibitor targeting programmed death 1, to conventional chemotherapy including carboplatin not only increased the pCR rate but also provided a longer event-free survival than the use of chemotherapy alone (31). Such positive result was obtained across all the subgroups of this study (e.g., with or without expression of programmed death ligand 1), which therefore supports the rational use of pembrolizumab among TNBC populations with diverse clinical characteristics in real-world settings. Apart from trying to achieve meaningful pCR, some drugs such as capecitabine and olaparib can still be applied to rescue non-pCR patients after neoadjuvant treatment if the prerequisite clinical conditions are fulfilled (26, 27).

### Potential study limitations

This study has some potential limitations. First, patient self-paid medications were not analyzed because this information was not available in our study administrative dataset (i.e., NHIRD). Some neoadjuvant drugs such as nanoparticle albumin-bound paclitaxel, bevacizumab, and pertuzumab, which have been reported in the literature, are not reimbursed by Taiwan’s NHI program and thus the use of these drugs could not be ascertained in this study. However, there is no indication for the use of nanoparticle albumin-bound paclitaxel or bevacizumab for eBC in Taiwan, and the indication of pertuzumab for eBC was filed in August 2015. Therefore, the utilization of these neoadjuvant drugs may have less impact on our study findings. Second, because the recording of recurrent or metastatic status is not mandatory in the TCR, it is difficult to differentiate the primary malignancy, secondary malignancy, or even recurrent events. As a result, the event-free survival of patients was difficult to justify in our data and thus not analyzed in this study. Third, the relationship between survival and adjuvant therapy after surgery was not assessed. Considering treatment patterns after surgery, the escalation of treatment intensity may be more frequently observed in the non-pCR population in contrast to the de-escalation of treatment in the pCR population. Therefore, because the difference in survival outcomes between the pCR and non-pCR populations is statistically apparent, providing detailed information of adjuvant treatments offers a trivial benefit. Fourth, details of patients’ treatment plan for neoadjuvant therapy (e.g., regimens, schedules) were not available in our administrative claims database. Therefore, additional information on the completion or tolerance of neoadjuvant treatment regimen among study populations were unable to be demonstrated or analyzed. Lastly, the generalizability of our study findings might be limited to Asian settings with national health insurance programs. However, the treatments that are reimbursed in the health insurance setting in our study reflect standard care in the most health care systems in developing countries. Therefore, our findings may be of value for real-life decisions in routine care settings.

## Conclusions

Achieving pCR was confirmed to be an important goal for better survival outcomes in HER2+ eBC and TNBC populations. Trastuzumab therapy provided HER2+ eBC patients a better chance of achieving pCR and overall survival. However, the use of platinum agents in TNBC settings only increased pCR rates; it did not further contribute to better survival outcomes. Therefore, optimal use of anti-HER2 target therapy in HER2+ eBC populations and the urgent need for novel therapy for TNBC should be considered in clinical treatment and health care policy decisions.

## Data availability statement

The datasets presented in this article are not readily available due to the data privacy and protection policy of the Health and Welfare Data Science Center. Requests to access the datasets should be directed to the corresponding author/s.

## Ethics statement

This study was approved by the Institutional Review Board of National Cheng Kung University (A-EX-106-029). Written informed consent for participation was not required for this study in accordance with the national legislation and the institutional requirements.

## Author contributions

Conception and design: W-PC, S-RY, C-TY, C-YS, H-WS, S-YL, H-TO. Analysis and interpretation of the data: W-PC, C-TY, H-TO. Drafting of the article: W-PC, C-TY, H-TO. Critical revision of the article for important intellectual content: W-PC, C-TY, H-TO. Provision of study materials or patients: H-TO. Statistical expertise: C-TY, H-TO. Administrative, technical, or logistic support: C-YS, H-WS, S-YL, H-TO. All authors contributed to the article and approved the submitted version.
