# Supplementary material for: Real-life analysis of neoadjuvant-therapy-associated benefits for pathological complete response and survival in early breast cancer patients - role of trastuzumab in HER2+ BC and platinum in TNBC
Source: Front Oncol. 2023 Jan 24;12:1022994. doi: 10.3389/fonc.2022.1022994 (PMC9902925; doi:10.3389/fonc.2022.1022994)
Supplement: Supplementary file 1 [file DataSheet_1.pdf]

Contents of tables, figures, supplementary tables, and supplementary figures

Supplementary Table 1: Baseline patient characteristics of HER2+ breast cancer patients stratified by status of pCR and exposure to trastuzumab

Supplementary Table 2: Baseline patient characteristics of TNBC patients stratified by status of pCR and exposure to platinum

Supplementary Figure 1: Exposure to individual chemotherapy agents in HER2+/LN+ and TNBC/LN+ patients

Supplementary Figure 2: Utilization of trastuzumab as neoadjuvant treatment agent among HER2+ patients with clinically positive lymph-node status during 2011-2016

Supplementary Figure 3: Utilization of platinum as neoadjuvant treatment agent among TNBC patients with clinically positive lymph-node status during 2011-2016

Supplementary Figure 4: KM curves of OS stratified by exposure to trastuzumab in HER2+/lymph-node-positive patients with pCR

Supplementary Figure 5: KM curves of OS stratified by exposure to trastuzumab in HER2+/lymph-node-positive patients without pCR

Supplementary Figure 6: KM curves of OS stratified by exposure to platinum in TNBC/lymph-node-positive patients with pCR

Supplementary Figure 7: KM curves of OS stratified by exposure to platinum in TNBC/lymph-node-positive patients without pCR

Supplementary Table 1: Baseline patient characteristics of HER2+ breast cancer patients stratified by status of pCR and exposure to trastuzumab (n=1,178)

	With pCR		Without pCR	
	**Trastuzumab (n=420)**	**No trastuzumab (n=40)**	**Trastuzumab (n=474)**	**No trastuzumab (n=244)**
**Age, years (mean ± SD)**	52.25 ± 10.14	51.25 ± 11.55	51.79 ± 10.53	51.57 ± 10.23
**Female (%)**	420 (100%)	40 (100%)	474 (100%)	243 (99.59%)
**Year of BC diagnosis (%)***				
2011	8 (1.90%)	12 (30.00%)	31 (6.54%)	54 (22.13%)
2012	33 (7.86%)	6 (15.00%)	43 (9.07%)	48 (19.67%)
2013	70 (16.67%)	5 (12.50%)	50 (10.55%)	37 (15.16%)
2014	83 (19.76%)	4 (10.00%)	105 (22.15%)	44 (18.03%)
2015	90 (21.43%)	7 (17.50%)	104 (21.94%)	40 (16.39%)
2016	136 (32.38%)	6 (15.00%)	141 (29.75%)	21 (8.61%)
**BR grade (%)**				
High	153 (36.43%)	12 (30.00%)	178 (37.55%)	104 (42.62%)
Low	80 (19.05%)	9 (22.50%)	98 (20.68%)	49 (20.08%)
Others	187 (44.52%)	19 (47.50%)	198 (41.77%)	91 (37.30%)
**Clinical stage (%)***				
Stage 2A	27 (6.43%)	2 (5.00%)	15 (3.16%)	5 (2.05%)
Stage 2B	158 (37.62%)	14 (35.00%)	174 (36.71%)	85 (34.84%)
Stage 3A	136 (32.38%)	15 (37.50%)	147 (31.01%)	76 (31.15%)
Stage 3B	44 (10.48%)	5 (12.50%)	68 (14.35%)	49 (20.08%)
Stage 3C	55 (13.10%)	4 (10.00%)	70 (14.77%)	29 (11.89%)
**Medical history**				
CCI = 0 (%)	298 (70.95%)	27 (67.50%)	361 (76.16%)	177 (72.54%)
CCI = 1 (%)	75 (17.86%)	9 (22.50%)	78 (16.46%)	47 (19.26%)
CCI ≥ 2 (%)	47 (11.19%)	4 (10.00%)	35 (7.38%)	20 (8.20%)

Abbreviations: pCR, pathological complete response; HER2, human epidermal growth factor receptor-2; BC, breast cancer; CCI, Charlson Comorbidity Index.
*Significant differences between patients with pCR and those without pCR (p<0.05)

Supplementary Table 2: Baseline patient characteristics of TNBC patients stratified by status of pCR and exposure to platinum (n=354)

	**With pCR**		**Without pCR**	
	**Platinum (n=61)**	**No platinum (n=67)**	**Platinum (n=62)**	**No platinum (n=164)**
**Age, years (mean ± SD)**	47.77 ± 10.24	48.93 ± 10.39	52.52 ± 10.97	51.48 ± 11.28
**Female (%)**	61 (100%)	67 (100%)	62 (100%)	164 (100%)
**Year of BC diagnosis (%)**				
2011	1 (1.64%)	6 (8.96%)	6 (9.68%)	21 (12.80%)
2012	8 (13.11%)	10 (14.93%)	9 (14.52%)	22 (13.41%)
2013	6 (9.84%)	9 (13.43%)	6 (9.68%)	15 (9.15%)
2014	8 (13.11%)	9 (13.43%)	10 (16.13%)	31 (18.90%)
2015	15 (24.59%)	12 (17.91%)	13 (20.97%)	38 (23.17%)
2016	23 (37.70%)	21 (31.34%)	18 (29.03%)	37 (22.56%)
**BR grade (%)**				
High	5 (8.20%)	14 (20.90%)	14 (22.58%)	37 (22.56%)
Low	27 (44.26%)	25 (37.31%)	31 (50.00%)	67 (40.85%)
Others	29 (47.54%)	28 (41.79%)	17 (27.42%)	60 (36.59%)
**Clinical stage (%)***				
Stage 2A	0 (0%)	4 (5.97%)	6 (9.68%)	2 (1.22%)
Stage 2B	29 (47.54%)	38 (56.72%)	10 (16.13%)	67 (40.85%)
Stage 3A	22 (36.07%)	15 (22.39%)	21 (33.87%)	55 (33.54%)
Stage 3B	5 (8.20%)	1 (1.49%)	10 (16.13%)	23 (14.02%)
Stage 3C	5 (8.20%)	9 (13.43%)	15 (24.19%)	17 (10.37%)
**Medical history**				
CCI = 0 (%)	51 (83.61%)	50 (74.63%)	47 (75.81%)	120 (73.17%)
CCI = 1 (%)	7 (11.48%)	16 (23.88%)	12 (19.35%)	27 (16.46%)
CCI ≥ 2 (%)	3 (4.92%)	1 (1.49%)	3 (4.84%)	17 (10.37%)

Abbreviations: pCR, pathological complete response; TNBC, triple-negative breast cancer; BC, breast cancer; CCI, Charlson Comorbidity Index.

*Significant differences between patients with pCR and those without pCR (p<0.05)

Supplementary Figure 1: Exposure to individual chemotherapy agents in HER2+/LN + and TNBC/LN+ patients

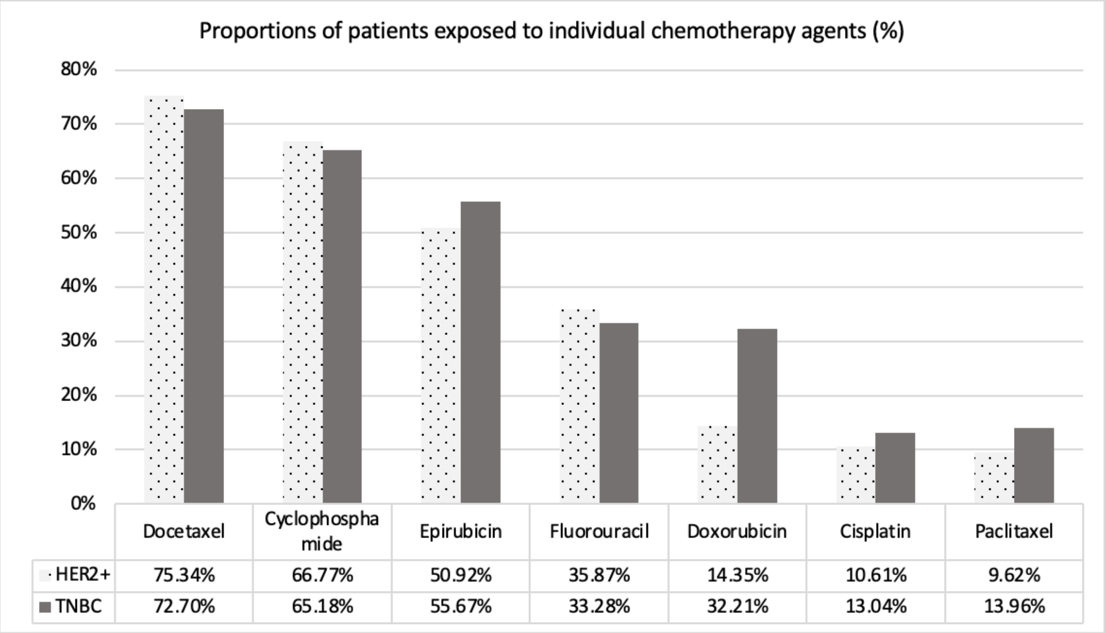

Abbreviations: HER2, human epidermal growth factor receptor-2; LN+, lymph-node-positive; TNBC, triple-negative breast cancer.

Note: Only the chemotherapy agents with higher than or equal to 5% of study patients are presented.

Supplementary Figure 2: Utilization of trastuzumab as neoadjuvant treatment agent among HER2+ patients with clinically positive lymph-node status during 2011-2016

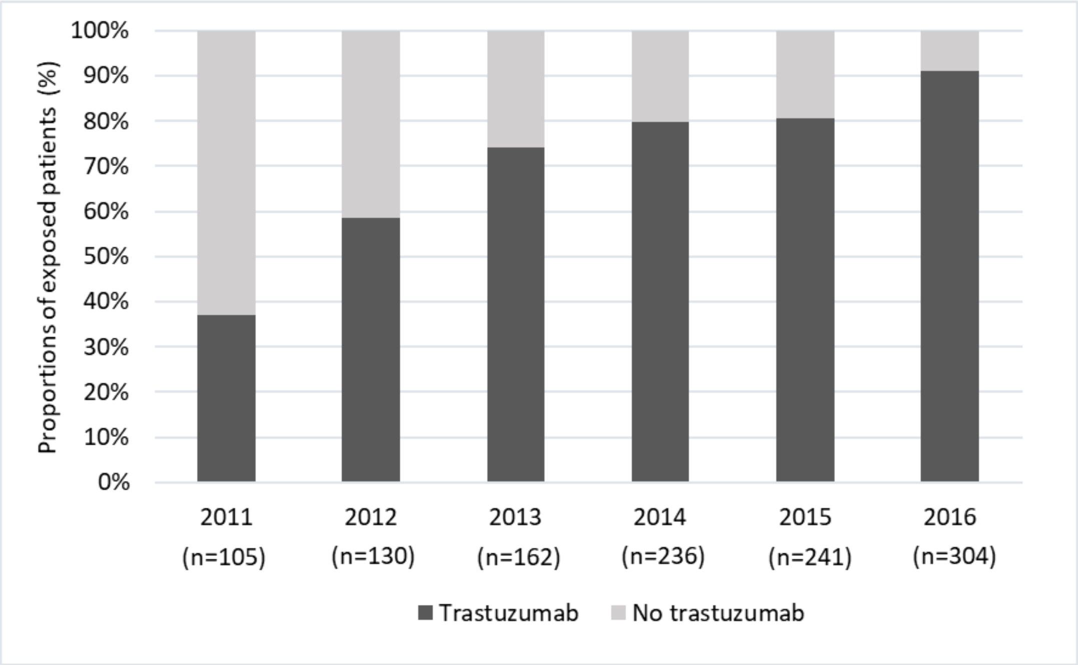

Supplementary Figure 3: Utilization of platinum as neoadjuvant treatment agent among TNBC patients with clinically positive lymph-node status during 2011-2016

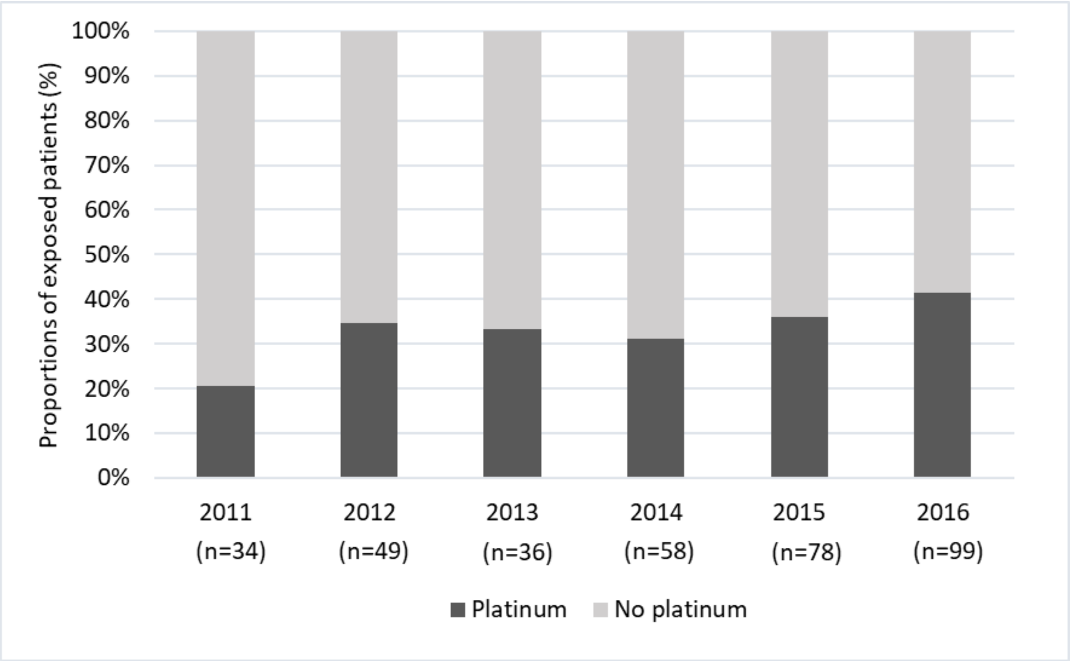

Supplementary Figure 4: KM curves of OS stratified by exposure to trastuzumab in HER2+/lymph-node-positive patients with pCR

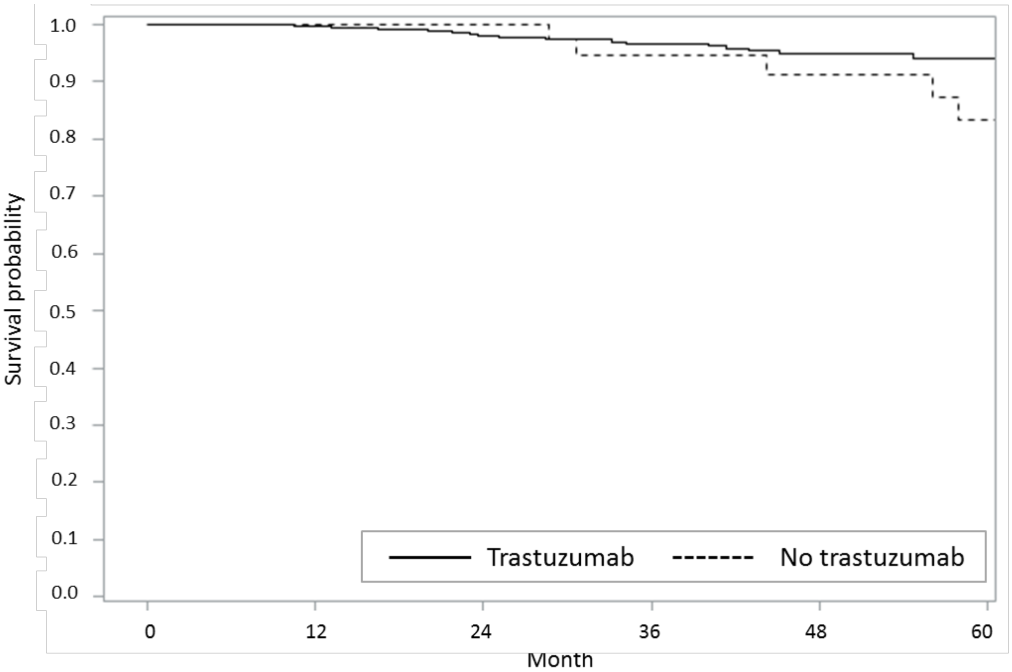

Patients at risk (n)

	0	12	24	36	48	60
—— Trastuzumab	420	419	412	273	183	109
--- No trastuzumab	40	40	40	32	24	19

Abbreviations: KM, Kaplan-Meier; OS, overall survival; HER2, human epidermal growth factor receptor-2; pCR, pathological complete response.

Supplementary Figure 5: KM curves of OS stratified by exposure to trastuzumab in HER2+/lymph-node-positive patients without pCR

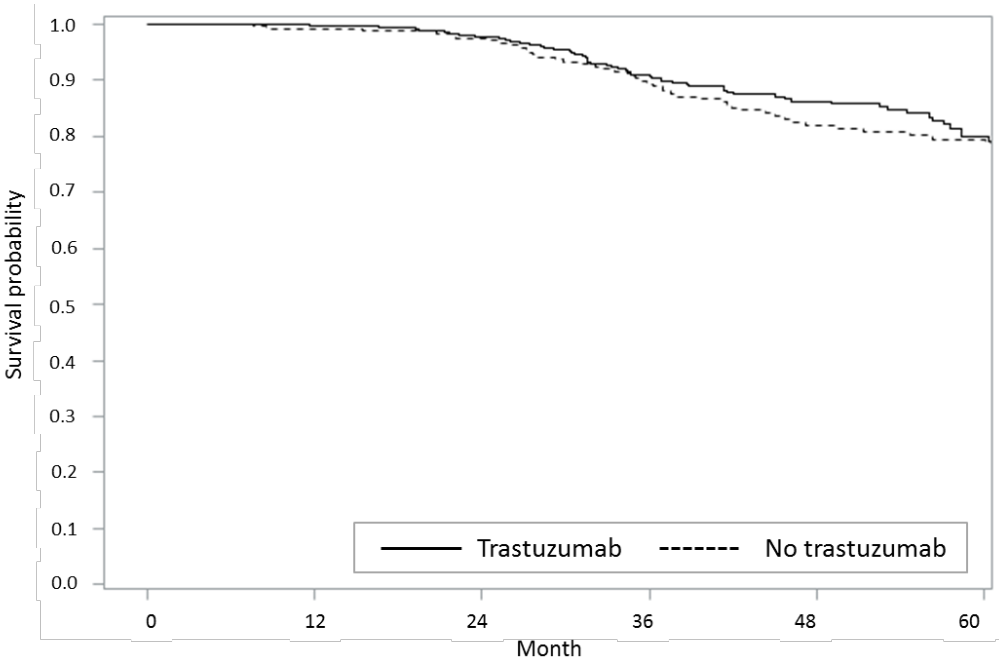

Patients at risk (n)

	0	12	24	36	48	60
—— Trastuzumab	474	473	463	304	196	103
- - - No trastuzumab	244	242	238	200	148	109

Abbreviations: KM, Kaplan-Meier; OS, overall survival; HER2, human epidermal growth factor receptor-2; pCR, pathological complete response.

Supplementary Figure 6: KM curves of OS stratified by exposure to platinum in TNBC/lymph-node-positive patients with pCR

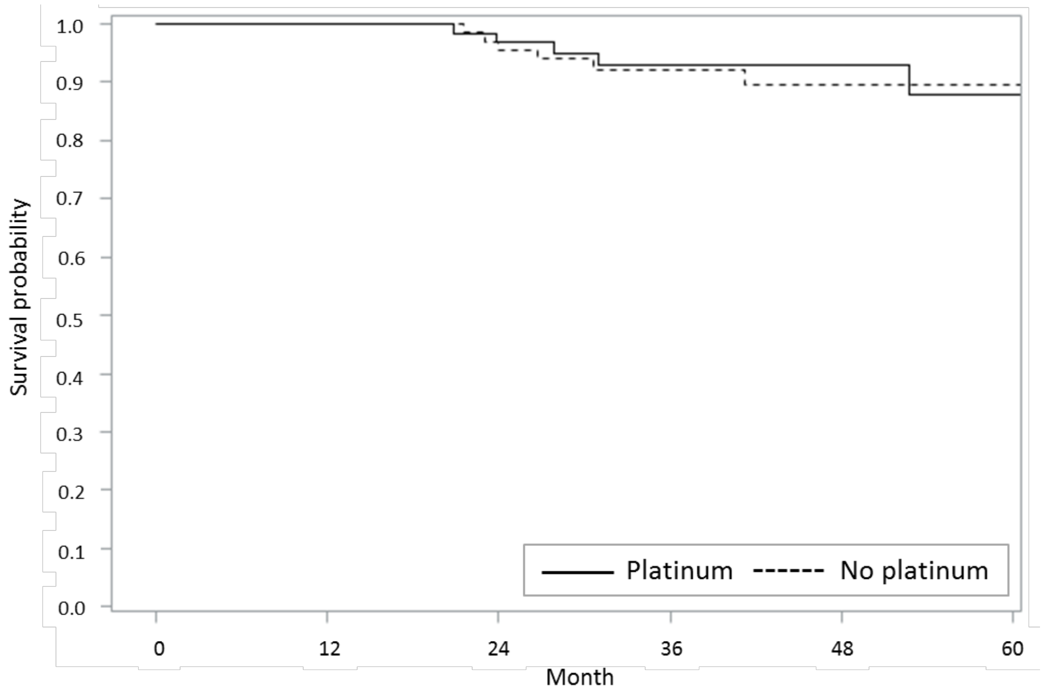

Patients at risk (n)						
Platinum	61	61	59	35	22	14
No platinum	67	67	64	42	30	23

Abbreviations: KM, Kaplan-Meier; OS, overall survival; TNBC, triple-negative breast cancer; pCR, pathological complete response.

Supplementary Figure 7: KM curves of OS stratified by exposure to platinum in TNBC/lymph-node-positive patients without pCR

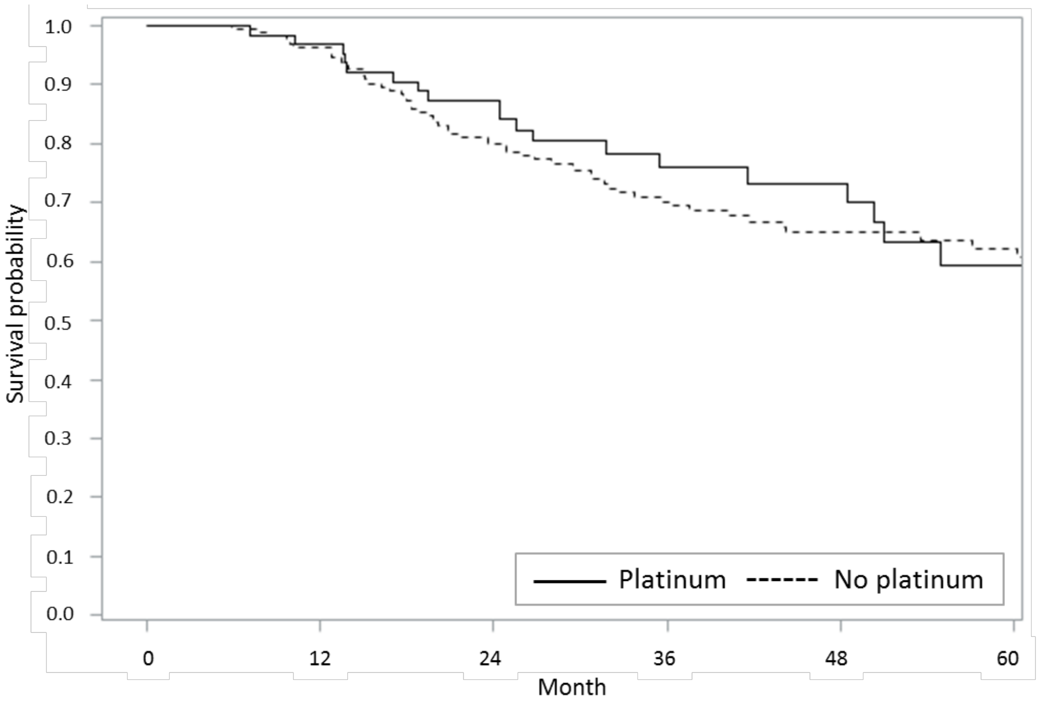

Patients at risk (n)

Platinum	62	60	54	31	23	12
No platinum	164	158	131	90	59	41

Abbreviations: KM, Kaplan-Meier; OS, overall survival; TNBC, triple-negative breast cancer; pCR, pathological complete response.
